# The Tissue Engineering Revolution: From Bench Research to Clinical Reality

**DOI:** 10.3390/biomedicines12020453

**Published:** 2024-02-18

**Authors:** Francesco De Chiara, Ainhoa Ferret-Miñana, Juan M. Fernández-Costa, Javier Ramón-Azcón

**Affiliations:** 1 Institute for Bioengineering of Catalonia (IBEC), The Barcelona Institute of Science and Technology (BIST), 08028 Barcelona, Spain; aferret@ibecbarcelona.eu (A.F.-M.); jramon@ibecbarcelona.eu (J.R.-A.); 2 ICREA-Institució Catalana de Recerca i Estudis Avançats, 08010 Barcelona, Spain; jfernandez@ibecbarcelona.eu

At its core, tissue engineering involves the use of a scaffold for the formation of new viable tissue for medical purposes. This field has expanded to include the development of biomaterials, cellular therapies, and the combination of both, with the integrative use of bioactive molecules to create not just replacements, but living, functional tissues that integrate with the patient’s body with unprecedented compatibility [1].

By examining the clinical applications of tissue engineering, this Special Issue brings to light the direct impact of tissue engineering research on patient care. It showcases the journey of innovations in this area from experimental studies to real-world healthcare solutions, underscoring the significance of interdisciplinary research in pushing the boundaries of what is medically feasible [2].

In the following sections, we will delve into a curated selection of studies, analyzing their methodologies, key findings, and broader implications for the field. By synthesizing their outcomes, we will assess the current landscape of tissue engineering, its recent advancements, and the challenges and opportunities that lie ahead. This introduction serves as the gateway to understanding the transformative potential of tissue engineering and sets the stage for a detailed exploration of its role in shaping the future of medicine.

Each study selected for this Special Issue has been chosen for its exemplary contribution to the field.

The diligent efforts of Feier et al. stand out at the forefront of personalized medicine, illuminating the methodology for the isolation of human mesenchymal stem cells (MSCs), a cornerstone of tailored orthopedic treatments and implant development [3]. This pursuit of customization in patient care is echoed in research that has successfully employed cultured autologous corneal epithelia (CACE) as a promising treatment for unilateral limbal stem cell deficiency (LSCD), showcasing the potential of cell-based therapies to precisely address individual patient needs [4].

Our focus now shifts towards treatment protocols and biomedical materials. In this regard, a study in this Special Issue evaluates the effectiveness of antiseptic treatments on bioengineered autologous skin substitutes (BASSs), which play a vital role in advancing burn care protocols [5]. This theme of material innovation extends into cardiovascular territory, where a comprehensive review sheds light on the application of tissue-engineered vascular grafts (TEVGs), demonstrating the possibility of transformative future treatments for cardiovascular diseases [6]. Similarly, the potential use of polymer nanocarriers in targeted drug delivery represents a pivotal step towards refining the efficacy of statin therapy, which indicates the broader movement towards targeted and material-based interventions [7].

Furthering the cause, the development of tissue surrogates, particularly for pulmonary lesions, forms a bridge between conceptual models and clinical applications, allowing for safer and more effective radiofrequency ablation techniques [8]. This emphasis on applied research is shared by a study utilizing the CUBIC technique for the 3D analysis of melanoma, enhancing our understanding of the tumor microenvironment and offering strategic insights to combat this formidable disease [9].

Within the cellular and molecular arena, we find studies that unravel the complex interactions between disease and treatment. The investigation into TGF-β2 and Dexamethasone treatments that improve trabecular meshwork cells’ resistance to mechanical stress lays the groundwork for novel glaucoma therapies [10]. Similarly, research revealing the interplay between fatty hepatocytes and skeletal muscle cells, as well as albumin’s protective role, offers hope for more effective treatments for fatty liver disease and it related muscle atrophy [11]. Complementing these works, a study probing the synergistic potential of fibrin, bone marrow cells, and macrophages in cardiac repair opens new avenues for therapeutic strategies in heart disease, indicative of the intricate dance between cellular components and therapeutic outcomes [12].

Woven together, these studies form a tapestry that depicts the present landscape and future horizons of tissue engineering. They collectively underscore a commitment to precision, patient-specific outcomes, and a deep-rooted belief in the power of interdisciplinary innovation to overcome the most daunting of clinical challenges.

For instance, one study might explore the use of biodegradable scaffolds in the regeneration of bone tissue. The methodology of such a study would likely involve the creation of a scaffold material that mimics the natural environment of bone cells to encourage growth and integration. Its findings might show promising results in the form of successful implantation in small mammal models, with key implications for the treatment of human bone fractures and defects.

When these individual studies are viewed collectively, a narrative begins to emerge—one of progress, challenges, and potential. The synthesis of these results not only confirms the viability of various tissue engineering strategies, but also reflects a growing understanding of the body’s complex biological processes [13,14]. For example, the collective outcomes from studies focusing on scaffold-based tissue regeneration reinforce the concept that the microenvironment is critical to tissue repair and that scaffolds must be designed with the precise biochemical and mechanical properties necessary for specific tissues [15,16,17].

Similarly, studies utilizing stem cells underscore the possibility of creating patient-specific treatments with a reduced risk of rejection and high therapeutic efficacy [18,19]. Collective evidence suggests that stem cells will become a cornerstone of regenerative medicine, with the capability to repair or replace a variety of damaged tissues, from neuronal to cardiac [20,21,22].

The combined results of these studies paint an encouraging picture for tissue engineering. They indicate a trend toward more sophisticated, personalized treatments, with a focus on mimicking natural tissue properties for better integration and function. This synthesis not only validates the methods used but also sets a benchmark for the quality and direction of future research.

The true measure of any scientific endeavor is its tangible impact on society, particularly in the domain of healthcare, where advances directly translate into enhanced patient care and outcomes. Tissue engineering, with its innovative approaches, stands at the precipice of revolutionizing clinical practice [23,24].

The clinical relevance of tissue engineering is as broad as it is profound. The studies discussed in this issue underscore this relevance, bringing forth a vision of healthcare that is more responsive, effective, and personalized. For instance, the advent of 3D-printed tissue structures has opened the door to custom implants tailored to an individual patient’s anatomy, thereby improving the success rates of surgical interventions and reducing recovery times. This is not merely theoretical; several studies have already demonstrated the successful implantations of 3D-printed tissues in patients, marking a significant step forward from laboratory to bedside [25].

Another example of clinical impact is the use of tissue-engineered skin for burn victims. Advances in this area have led to the development of bi-layered skin grafts that not only cover wounds but also promote healing and reduce scarring. The key findings from these studies have been integrated into protocols for burn treatments, illustrating a direct line from research to clinical application.

The implications for patient outcomes are just as compelling. Tissue engineering strategies have the potential to reduce occurrences of transplant rejection through the use of the patient’s own cells to grow tissues or organs. This could significantly diminish patients’ long-term reliance on immunosuppressive drugs and their associated side effects, thereby improving the quality of life for transplant recipients [26,27].

Moreover, the field is making strides forward in regenerative medicine, particularly in the repair of damaged cardiac tissue following myocardial infarction. The utilization of tissue-engineered cardiac patches has been linked to improved heart function in preclinical studies, indicating its potential to reduce morbidity and mortality in heart disease patients.

The findings from this cutting-edge research not only advocate for the integration of tissue engineering solutions into current medical practice but also lay the groundwork for a future in which regenerative treatments are commonplace. The promise of reduced healthcare costs, alongside the improvement in patient quality of life, propels this field; not just as a scientific curiosity but as a pivotal element of clinical strategy moving forward.

The dynamic field of tissue engineering is ever-evolving, with each year bringing new breakthroughs that push the boundaries of what is possible in regenerative medicine. These advancements not only reflect the ingenuity of the researchers but also promise to address some of the most challenging medical conditions we face today.

One of the most striking recent developments in tissue engineering is the use of gene-editing technologies, like CRISPR/Cas9, to create scaffolds that can actively participate in the healing and regeneration of tissues [28]. These scaffolds are no longer inert structures but are now being designed to release growth factors or genetic material at the site of injury, thereby enhancing tissue integration and function.

Another notable advancement is the emergence of organ-on-a-chip technology, which involves creating tiny models of human organs on microchips. These chips mimic the complex biological functions of specific organ systems and are revolutionizing drug testing and disease modeling. This technology could drastically reduce the need for animal testing and provide a more accurate representation of human physiology, leading to better patient outcomes [29].

When these cutting-edge advancements are juxtaposed with the findings contained in this Special Issue, both convergences and divergences come to light. For example, the use of biodegradable scaffolds for bone regeneration, as highlighted in earlier studies, laid the groundwork for the gene-edited scaffolds of today. The principles remain the same—facilitating tissue growth and integration—but the methods have become more sophisticated, precise, and personalized.

Conversely, while organ-on-a-chip technology represents a leap forward in the field, it also underscores a gap that exists in current tissue engineering applications: the transition from two-dimensional to three-dimensional complexity. The studies in this Special Issue predominantly focus on tissue repair and regeneration, while organ-on-a-chip models are a foray into the intricate emulation of whole organ systems, highlighting a new horizon for tissue engineering research and its potential impact on clinical practices.

These recent developments, when analyzed alongside this Special Issue’s findings, reflect both an evolution of established techniques and the advent of entirely novel approaches in the field of tissue engineering. This comparative analysis not only acknowledges the remarkable progress made so far, but also emphasizes the need for continued innovation and exploration to overcome current limitations and fully realize the clinical potential of tissue engineering.

As with any field balanced on the cutting edge of science and technology, tissue engineering faces its share of challenges. These obstacles not only test the resilience and creativity of researchers, but also define the trajectory of future advancements in the field.

One of the most significant challenges in tissue engineering is the replication of complex tissue structures and functions, particularly for organs with high cellular heterogeneity and intricate architectures, such as the liver or kidneys [30]. While scaffold technology and stem cell therapy have shown promise, achieving the level of precision necessary for these organs remains a daunting task. Vascularization, or the process of forming blood vessels within engineered tissues, is another hurdle [31]. Without proper blood flow, even the most meticulously engineered tissues cannot survive, let alone function, after transplantation.

Moreover, the regulatory landscape adds another layer of complexity. Ensuring that new tissue engineering products are safe and effective requires navigating a maze of approval processes, which can be both time-consuming and costly. This is a necessary challenge, however, as it ensures patient safety and the efficacy of treatments.

Despite these challenges, the future of tissue engineering is brimming with potential. The convergence of bioengineering, materials science, and digital technology is paving the way for novel approaches. For instance, advancements in bioprinting technologies promise the ability to construct tissues layer-by-layer, potentially overcoming current vascularization issues.

Another exciting frontier is the development of smart biomaterials that can respond to their biological environment, providing cues for tissue healing or regeneration as needed. The integration of such materials into emerging technologies such as nano- and micro-technology could lead to the development of next-generation implants that can adaptively respond to a patient’s needs [32].

Moreover, with the growing emphasis on personalized medicine, tissue engineering is poised to offer more customized solutions. The use of patient-specific genetic information could allow for the engineering of tissues that are more compatible with an individual’s biology, reducing the risk of rejection and enhancing therapeutic outcomes.

In the broader scope of the field, interdisciplinary collaboration will be vital to overcoming current limitations. Combining insights from different scientific disciplines will not only accelerate the pace of innovation but also enhance the translatability of bench research into clinical applications. As researchers continue to untangle the complexities of human tissues, the day when organ shortages and irreparable tissue damage are problems of the past edges closer to reality.

As we begin to conclude our exploration of this Special Issue dedicated to tissue engineering, it is clear that the contributions of these studies are both foundational and transformative within the field of biomedicine.

The collected works provide a comprehensive snapshot of the current state of tissue engineering. From the detailed methodologies that guide tissue scaffold development to the clinical trials that bring these innovations to the forefront of patient care, each study contributes a vital piece to the larger puzzle. The research summarized herein not only advances our scientific understanding but also serves as a beacon for future explorations, setting new standards for what can be achieved in regenerative medicine.

The clinical impacts discussed reflect a growing integration of tissue engineering solutions into healthcare, with the potential for improved outcomes for patients across a spectrum of conditions. Advancements in the field, including the groundbreaking applications of gene editing and organ-on-a-chip technologies, are the leaps forward that are propelling tissue engineering from a concept to a clinical reality.

Looking forward, the trajectory of tissue engineering is set to reshape clinical settings in profound ways. With each barrier that is overcome, the promises of this field—once considered futuristic—are being realized, offering hope that personalized and effective treatment options are not far. The challenges that remain, while significant, provide a roadmap for researchers and clinicians alike, pointing towards a horizon rich with possibility.

As the dialogue between research and clinical practice continues to evolve, so too will the strategies and applications of tissue engineering. This Special Issue stands not just as a testament to the achievements made thus far, but also as an invitation to the scientific community to continue pushing the boundaries of what can be healed, repaired, and improved upon in the human body.

In closing, the collective insights garnered from these studies underscore a pivotal era in tissue engineering. The future is one where the regeneration of complex tissues and organs is not just a possibility but a reality, transforming the landscape of medicine and patient care for generations to come. Building on this vision, the direction of future tissue and regenerative medicine research is set to leverage groundbreaking technologies such as CRISPR/Cas9 for gene editing and organ-on-a-chip models. These advancements promise to further enhance tissue integration and function, offering more accurate physiological representations and improving patient outcomes. As we continue to navigate the complexities of human tissue regeneration, these innovations offer a glimpse into a future where personalized and effective treatment options are not just a goal but a reality. The journey ahead is rich with potential, inviting the scientific community to explore the uncharted territories of regenerative medicine and redefine what is possible in healthcare. 
